# Supplementary material for: Mutations in PCYT2 disrupt etherlipid biosynthesis and cause a complex hereditary spastic paraplegia
Source: Brain. 2019 Oct 22;142(11):3382–97. doi: 10.1093/brain/awz291 (PMC6821184; doi:10.1093/brain/awz291)
Supplement: awz291_Supplementary_Data [file awz291_supplementary_data.zip › awz291-Suppl_data/Supplementary_Data4.zip!/Supplementary information 3 - fibroblast lipidomics.zip!/Fibroblast lipidomics/Controle vs Patient2/Statistical plots and data/PCA.pdf]

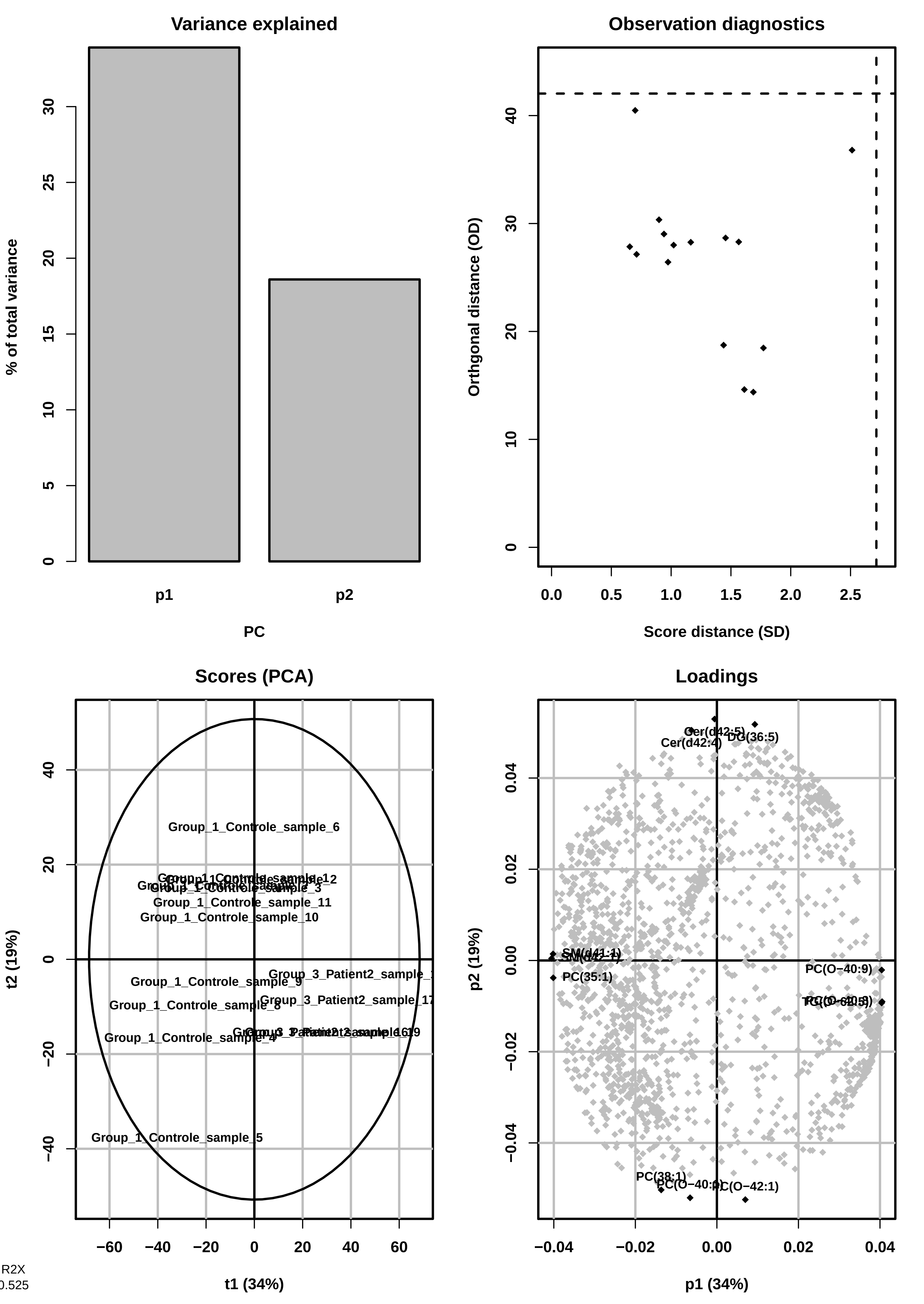
Variance explained
% of total variance
PC
p1
p2
Observation diagnostics
Orthgonal distance (OD)
Score distance (SD)
Scores (PCA)
t1 (34%)
t2 (19%)
Group_1_Controle_sample_6
Group_1_Controle_sample_11
Group_1_Controle_sample_10
Group_1_Controle_sample_9
Group_1_Controle_sample_8
Group_1_Controle_sample_4
Group_1_Controle_sample_5
Group_3_Patient2_sample_17
Loadings
p1 (34%)
p2 (19%)
Cer(d42:5)
Cer(d42:4)
DG(36:5)
SM(d41:1)
PC(35:1)
PC(O-40:9)
PC(38:1)
PC(O-42:1)
R2X
0.525
